# Effects of a Web-based Intervention on Cardiovascular and Physical Health of Korean Older Adults During the Pandemic

**DOI:** 10.1093/geroni/igab046.3379

**Published:** 2021-12-17

**Authors:** Soonhyung Kwon, Oejin Shin

**Affiliations:** 1 University of Illinois at Urbana-Champaign, Savoy, Illinois, United States; 2 University of Illinois at Urbana Champaign, Savoy, Illinois, United States

## Abstract

During the lockdown, 97.5% of Korean senior centers in South Korea were closed to prevent the spread of the coronavirus disease 2019 (COVID-19). The threat of the COVID-19 presented the need for alternative interventions for Korean older adults to maintain cardiovascular and physical health. Korean senior centers implemented web-based interventions to provide physical health services, but their effectiveness was not yet assessed. Thus, our study aimed to identify the effects of a web-based intervention using a smartwatch and mobile app in older adults when compared to center-based intervention during the pandemic. This study collected 117 Korean older adults (≥ 60) who participated in the 12-week web-based and center-based physical interventions using a smartwatch and mobile app. This quasi-experimental study was conducted between August and December in 2020. We analyzed the pre-posttest of cardiovascular and physical health across two intervention types. Our regression results indicated that participants in the 12-week web-based intervention reported better cardiovascular (systolic blood pressure: b = -13.77, p < .001; cholesterol: b = -11.71, p < .05) and physical health (muscular function: b = 2.99, p < .001; body balance: b = -1.31, p < .001; cardiopulmonary endurance: b = 33.33, p < .001) than those in center-based intervention at posttest. The findings imply a web-based intervention is likely to become an innovative therapeutic strategy for older adults' health to respond to the rapidly changing social service systems amid the pandemic.

